# Enterovirus D-68 Infection, Prophylaxis, and Vaccination in a Novel Permissive Animal Model, the Cotton Rat (*Sigmodon hispidus*)

**DOI:** 10.1371/journal.pone.0166336

**Published:** 2016-11-04

**Authors:** Mira C. Patel, Wei Wang, Lioubov M. Pletneva, Seesandra V. Rajagopala, Yi Tan, Tina V. Hartert, Marina S. Boukhvalova, Stefanie N. Vogel, Suman R. Das, Jorge C. G. Blanco

**Affiliations:** 1 Sigmovir Biosystems Inc., Rockville, Maryland, United States of America; 2 Department of Microbiology and Immunology, University of Maryland, Baltimore, Maryland, United States of America; 3 Infectious Diseases Group, J. Craig Venter Institute, Rockville, Maryland, United States of America; 4 Department of Medicine, Vanderbilt University Medical Center, Nashville, Tennessee, United States of America; Stanford University School of Medicine, UNITED STATES

## Abstract

In recent years, there has been a significant increase in detection of Enterovirus D-68 (EV-D68) among patients with severe respiratory infections worldwide. EV-D68 is now recognized as a re-emerging pathogen; however, due to lack of a permissive animal model for EV-D68, a comprehensive understanding of the pathogenesis and immune response against EV-D68 has been hampered. Recently, it was shown that EV-D68 has a strong affinity for α2,6-linked sialic acids (SAs) and we have shown previously that α2,6-linked SAs are abundantly present in the respiratory tract of cotton rats (*Sigmodon hispidus*). Thus, we hypothesized that cotton rats could be a potential model for EV-D68 infection. Here, we evaluated the ability of two recently isolated EV-D68 strains (VANBT/1 and MO/14/49), along with the historical prototype Fermon strain (ATCC), to infect cotton rats. We found that cotton rats are permissive to EV-D68 infection without virus adaptation. The different strains of EV-D68 showed variable infection profiles and the ability to produce neutralizing antibody (NA) upon intranasal infection or intramuscular immunization. Infection with the VANBT/1 resulted in significant induction of pulmonary cytokine gene expression and lung pathology. Intramuscular immunization with live VANBT/1 or MO/14/49 induced strong homologous antibody responses, but a moderate heterologous NA response. We showed that passive prophylactic administration of serum with high content of NA against VANBT/1 resulted in an efficient antiviral therapy. VANBT/1-immunized animals showed complete protection from VANBT/1 challenge, but induced strong pulmonary Th1 and Th2 cytokine responses and enhanced lung pathology, indicating the generation of exacerbated immune response by immunization. In conclusion, our data illustrate that the cotton rat is a powerful animal model that provides an experimental platform to investigate pathogenesis, immune response, anti-viral therapies and vaccines against EV-D68 infection.

## Introduction

Picornaviruses of the genus *Enterovirus* (EV) comprise many human pathogens that cause most common infections in humans, such as EV A-D and rhinovirus (RV) A-C [1]. The EVs are small, single-stranded, positive-sense RNA viruses with a genome of ~7.5 kb, encapsidated into an icosahedral capsid, forming a non-enveloped virion of around 30 nm diameter. There are total 5 types of EV-D species: EV-D70, associated with acute hemorrhagic conjunctivitis [2, 3], EV-D94, causative agent of acute flaccid paralysis [4, 5], EV-D111 and D120, identified in non-human primates [6, 7], and EV-D68. EV-D68 was first isolated from four hospitalized pediatric patients with pneumonia and bronchiolitis in California in 1962 [8], indicating that its initial tropism targets the respiratory tract. There are three major clades of EV-D68, designated as A, B and C, which are circulating worldwide [9, 10]. The EV-D68 genome consists of single open reading frame (ORF), encoding four structural proteins (VP1-VP4) and seven nonstructural proteins (2A-2C and 3A-3D), flanked by a long 5’ untranslated region (UTR) with a hairpin-loop secondary structure and a short 3’UTR with a poly(A) tract [11].

Since its discovery in 1962, EV-D68 infections were among the most rarely reported until the early 2000’s [12]. However, an upsurge in detection of EV-D68 has been documented in the last decade among patients with acute respiratory infections of various severities, ranging from mild upper respiratory tract infections to severe pneumonia, including fatalities in pediatric and adult patients [9–11, 13–22]. In 2014, the largest outbreak of EV-D68 infection in USA was reported. From mid-August 2014 to January 2015, a total of 1,153 cases of respiratory illness caused by EV-D68 in 49 states and in the District of Columbia were reported, which were confirmed by either the Centers for Disease Control and Prevention (CDC) or different State public health laboratories [23, 24]. Most cases were children, with a large percentage of them requiring pediatric intensive care, and some cases were fatal [25]. Previously, EV-D68 was detected in the cerebrospinal fluid (CSF) in a 5 year-old boy who died due to meningomyeloencephalitis and pneumonia [26]. During the 2014 USA outbreak, a geographically and temporally defined cluster of cases with acute flaccid paralysis and cranial nerve dysfunction was also reported in 12 children, where the virus was detected sporadically in nasopharyngeal samples [27]. In addition, 3 cases of pediatric EV-D68 infections associated with acute flaccid paralysis were also reported in Europe in 2014 [28, 29]. In 2016, a total of 50 cases of acute flaccid myelitis were confirmed in 24 states (cases reported up to August 31), while limited sporadic cases of EV-D68 have been detected across USA [30]. These reports have raised concerns that genetic changes in EV-D68 could be contributing to the increased detection of the virus in human respiratory infections and the increase in disease severity and neurological symptoms. Thus, EV-D68 is now recognized as a re-emerging pathogen [11]. Currently, there is no specific antiviral therapy against EV-D68 available and treatment is primarily supportive. Furthermore, until now, there has been no suitable animal model available to develop and test therapeutics against EV-D68 virus and to obtain comprehensive understanding of its pathogenesis.

Over the years, EV-D68 genome has undergone many deletions in the spacer region of the 5’ UTR between the end of the internal ribosome entry sites (IRES) and the polyprotein ORF. The significance of these deletions is not clear; however, such mutations might influence translational efficiency and thereby affect viral virulence. Clades A and B are further divided into subclades: A1 and A2 and B1 and B2, on the basis of amino acid substitutions in both structural and nonstructural proteins [11]. Compared to clades A and B, clade C is geographically restricted and circulated in Japan during 2005 to 2010 and in Italy during 2008 [9, 14, 17]. Subclades A1 and B2 are considered endemic and were found in many countries before the 2014 outbreak, such as Thailand from 2006 to 2011 [19], the United Kingdom from 2009 to 2010 [18], China from 2009 to 2012 [20], the Philippines from 2009 to 2014 [13, 15, 16], and the Netherlands from 1994 to 2014 [21, 22]. During the 2014 outbreak, subclade B1 was dominant among USA cases (specimens collected from Kansas City, MO) [31], while another report showed that the majority of EV-D68 strains circulating in the 2014 outbreak (specimens collected from the Lower Hudson Valley of New York) differ significantly from prior ones, mostly having three nucleotide variables, C1817T, C3277A and A4020G, and belong to a new clade [32].

Sialic acids (SAs) are receptors for EV-D68 [33, 34]. Using glycan array and enzymatically modified erythrocytes, it was shown that EV-D68 has a stronger affinity for α2,6-linked SAs than α2,3-linked SAs [33]. The SA receptor induces a cascade of conformational changes in the EV-D68 virus that prime viral uncoating and facilitate cell entry [34]. Lectin-based staining showed that both α2,3-linked and α2,6-linked SA receptors are present in the respiratory tract of cotton rats; α2,6-linked SA receptors were found on ciliated cells, whereas α2,3-linked SA receptors were more associated with mucin-producing cells in the cotton rat trachea. Cotton rat lung parenchyma showed a consistent staining of type I and type II pneumocytes with α2,6-linked SA, but undetectable levels of α2,3-linked SA [35]. Consistent with these observations, we have shown that influenza A of human and avian origin, as well as influenza B isolates replicate without the need for “adaptation” in cotton rat upper and lower respiratory tracts [35, 36]. In addition, we reported that intranasal infection of cotton rats with another picornavirus, human rhinovirus (HRV) type 16, resulted in isolation of infective virus, lower respiratory tract pathology, mucus production, and expression of interferon-activated genes without any genetic modification of either the host or the virus [37]. In contrast to other EVs, EV-D68 is biologically more similar to HRVs [38]. In fact, HRV87, discovered in 1963, was subsequently reclassified as EV-D68 based on molecular analysis [39, 40]. Similar to RVs, EV-D68 grows optimally at 33°C compared to 37°C preferred by other EVs, and is both heat and acid labile [38].

In the present study, we evaluated three strains of EV-D68, belonging to different clades, for their abilities to infect cotton rats. We report that intranasal (i.n.) infection of cotton rats with EV-D68 (VANBT/1) resulted in isolation of infective virus from the nose and lung tissues, expression of lung inflammatory cytokines, and marked lung pathology. Infection and immunization of cotton rats with live EV-D68 generated various levels of protection from virus challenge that correlated with the production of different levels of serum NA. Furthermore, we demonstrate that this model could be an excellent tool to decipher cross-reactive immunity among different EV-D68 clades, which is relevant for the generation of an efficacious EV-D68 vaccine with broad protection. We conclude that EV-D68 infection in cotton rats can provide novel insights that will enable the molecular dissection of immune responses to EV-D68 and thus develop effective intervention and prevention strategies.

## Results

### EV-D68 infection and replication in cotton rats

**Fig 1:**
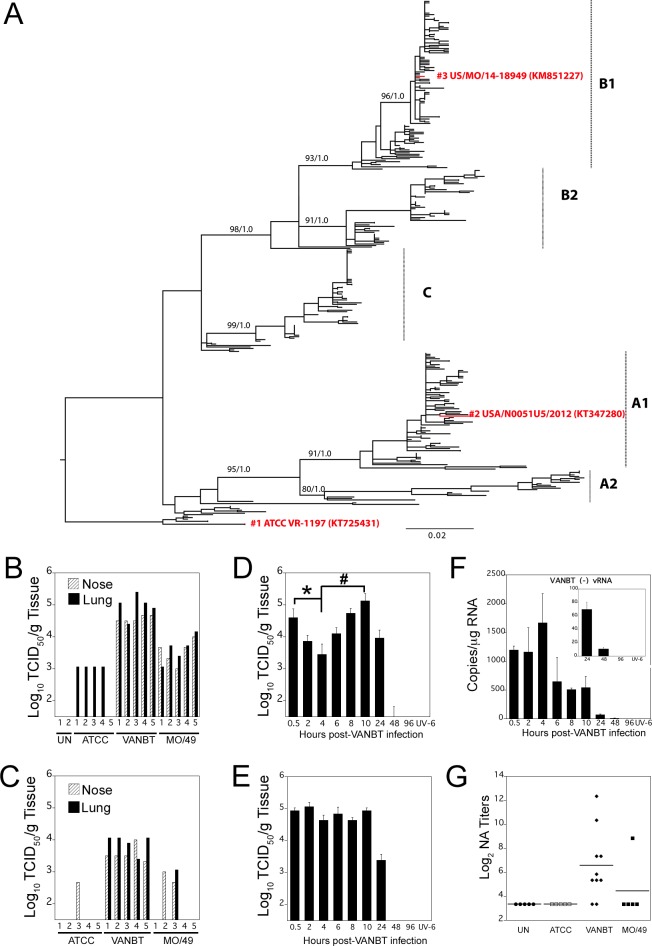
A cotton rat model of EV-D68 infection and replication. Cotton rats were infected i.n. with 10^6^ TCID_50_ per rat with 3 different strains of EV-D68, ATCC (prototype Fermon strain), VANBT (2012 pre-outbreak strain from Nashville, TN) and MO/49 (2014 outbreak strain from Kansas, MO). (A) Evolutionary tree showing three major clades of EV-D68, A, B and C, distributed worldwide. Strains used in this study are shown in red. The tree is rooted by the oldest EV-D68 sequence in GenBank, the Fermon strain (referred to as ATCC), collected in 1962 in California, USA. (B, C) Quantification of infectious virus titers of each EV-D68 strain in nose and lung homogenates from infected animals at 10 h (B) and 24 h (C) p.i. Groups of 5 animals were euthanized at each time point. Each bar corresponds to an individual animal. UN = uninfected. (D, E, F) Time course of VANBT replication in cotton rats; groups of 4 animals were euthanized at the indicated time to measure infectious virus titers in nose (D) and lung (E) homogenates. Results are representative of 2 independent experiments. *p<0.05 for 4 h nose titer compared with 0.5 h p.i. and #p<0.05 for 10 h nose titer compared with 4 h p.i. Animals inoculated with UV-VANBT and sacrificed at 6 h p.i. (UV-6) were shown as control. (F) Quantification of VANBT (-) vRNA by qRT-PCR in lung tissue. Insert is a blowup of the 24, 48 and 96 h time points from VANBT-infected and UV-VANBT-inoculated rat sacrificed at 6 h p.i. (G) Homologous NA titers measured in serum samples collected 3 weeks p.i. with the indicated EV-D68.

For this work, three different strains of EV-D68 were used to encompass both clades A and B, representing their historical appearance relevant to USA. We classified the three strains as: (1) classical ATCC (Accession # KT725431, referred as ATCC), which is the prototype Fermon virus strain purchased from ATCC. The Fermon strain is the oldest EV-D68 sequence in GenBank and it was collected in 1962 in California [8]. The sequence of the ATCC strain is clustered near the root of phylogenetic tree, reflecting its sampling date in the 1960s (Fig 1A). (2) Pre-outbreak isolate VANBT/1 (Accession # KT347280, referred to as VANBT) was collected in 2012 from Nashville, TN at Vanderbilt University Medical Center, and belongs to subclade A1 (Fig 1A); and, (3) outbreak isolate MO/14/49 (Accession # KM851227, referred to as MO/49), was collected in Kansas City, MO during the 2014 outbreak, and belongs to subclade B1 (Fig 1A). All the three strains were propagated in HeLa H1 cells at 34°C and their titers were determined by standard endpoint dilution assay and expressed as TCID_50_/ml.

We infected groups of 10 adult cotton rats i.n. with 10^6^ TCID_50_ of each of the 3 strains of EV-D68. Five animals per group were euthanized at 10 and 24 h post infection (p.i.) for determination of viral titers in the nose and lung of each animal (Fig 1B and 1C). ATCC virus was recovered from the lung, but not from the nose at 10 h p.i., whereas detection of this strain was almost negligible in both tissues at 24 h p.i. (only one animal exhibited virus in the nose). VANBT was consistently recovered from both nose and lung tissues at 10 and 24 h p.i. The amount of VANBT virus recovered was generally higher in the lung than in the nose at both 10 and 24 h p.i. (Fig 1B and 1C). The decrease in recovered infectious VANBT titer was around ~1 log_10_ in both the tissues at 24 h p.i. compared to 10 h. MO/49 titers in both nose and lung tissues were comparable among all 5 animals at 10 h p.i. (3.5 to 4 log_10_ TCID_50_/g tissue), but isolation was less consistent at 24 h p.i. (Fig 1B and 1C). Overall, these results show that VANBT replicates more strongly than MO/49 or ATCC in cotton rats and, thus, we used VANBT for subsequent infection experiments.

Adult cotton rats were infected i.n. with 10^6^ TCID_50_ of VANBT or inoculated i.n. with an identical amount of UV-inactivated VANBT virus (UV-VANBT). Groups of 4 animals were euthanized at 0.5, 2, 4, 6, 8, 10, 24, 48, and 96 h p.i. to measure the profile of virus replication in nose and lung tissues (Fig 1D and 1E, respectively). VANBT virus was detected in the nose at 0.5 h, which showed a brief but defined drop at 2–4 h p.i. (*p<0.05 for 4 h compared to 0.5 h p.i. titer) that corresponds to the viral eclipse, and a subsequent increase at 6 h, reaching a peak in this tissue at 10 h p.i. (#p<0.05 for 10 h compared with 4 h p.i. titer) (Fig 1D). Infectious virus was almost undetectable at 48 h p.i. in the nose. In the lung, virus titers remained constant between 0.5 to 10 h p.i. and subsequently decreased to undetectable levels by 48 h (Fig 1E). As expected, no infectious virus was detected in the lung and nose of animals inoculated with UV-VANBT (Fig 1D and 1E, UV-6). To determine whether there was a sex-bias in the replication of EV-D68 in cotton rats, we compared virus yields at 10 h p.i. in nose and lung tissues from both male and female cotton rats. VANBT titers recovered from these tissues were essentially identical between animals of different sexes (S1 Fig).

For VANBT, the extent of viral replication was also assessed by determining the amount of negative strand viral RNA [(-) vRNA] (replication intermediates) by qRT-PCR. (-) vRNA strands were detected in lungs of infected animals up to 48 h p.i. (Fig 1F, and insert). The levels of (-) vRNA in the lung briefly peaked at 4 h, maintained a brief plateau between 6–10 h, followed by a decrease and final clearance of viral intermediate by 96 h p.i. Similar to (-) vRNA, quantification of total VANBT vRNA by qRT-PCR showed an almost comparable pattern (S2 Fig). Animals inoculated with UV-VANBT showed undetectable levels of (-) vRNA and total vRNA (Fig 1F and S2 Fig).

A set of animals infected i.n. with the different EV-D68 strains (ATCC, VANBT, and MO/49) were followed 21 days after challenge to determine whether infection by the three strains induced immunologic responses in the form of NA titer. Paralleling their various levels of replication in cotton rats, VANBT infection generated the highest NA titer (8 of 10 infected animals showed detectable homologous NA titers), MO/49 infection showed only one animal with detectable NA titer, and ATCC infection resulted in no detectable NA response (Fig 1G). Moreover, the NA titer against VANBT persisted with the comparable strength through 9 weeks p.i. (S3 Fig).

### Induction of pulmonary cytokines and lung pathology in response to VANBT infection

**Fig 2:**
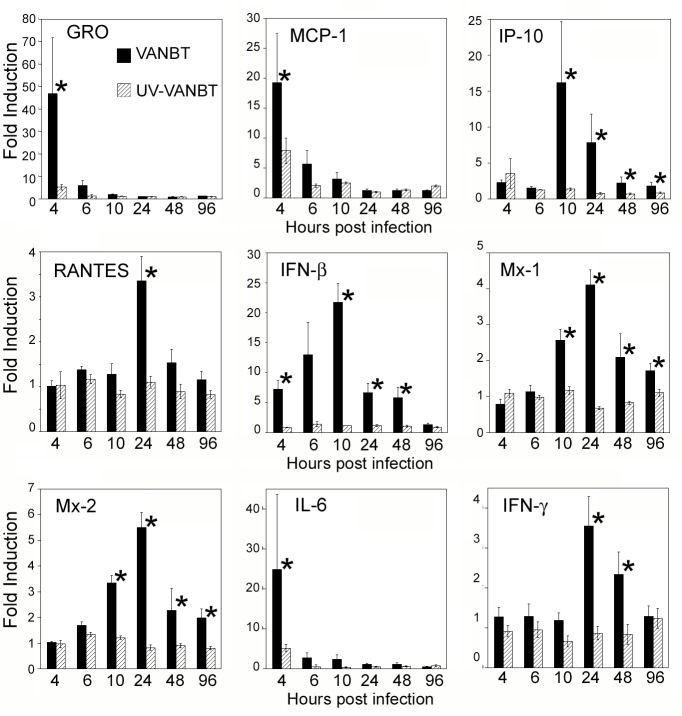
VANBT infection in cotton rats induces pulmonary chemokine, IFN, and proinflammatory cytokine mRNA expression. Cotton rats were infected i.n. with 10^6^ TCID_50_ of VANBT or the same amount of UV-VANBT. Groups of 4 animals were euthanized at each indicated time point. Results are representative of two independent experiments. Relative gene expression profile of chemokines (GRO, MCP-1, IP-10 and RANTES), IFN-β, two IFN-inducible genes (Mx-1 and Mx-2), and pro-inflammatory cytokines (IL-6 and IFN-γ) in the lung tissues were measured by qRT-PCR. Results are calculated as fold induction over uninfected (naïve) animals and expressed as geometric means ± SE, *p<0.05 for VANBT compared with UV-VANBT at each time point.

To determine effect of the EV-D68 infection on the induction of an inflammatory response, we focused on the lung tissue and measured the expression of cotton rat mRNA for several chemokines, Type I and Type II interferons (IFNs), cytokines, and select IFN-inducible genes following VANBT infection. Cotton rats were infected with VANBT or UV-VANBT and euthanized at the indicated time points to measure levels of gene expression by qRT-PCR (Fig 2). VANBT infection dramatically induced early production of the neutrophil chemoattractant chemokine GRO and monocyte chemotactic protein 1 (MCP-1), peaking within 4 h and sharply decreasing to basal levels by 10 h of infection. A different profile was found for chemokines IP-10 and RANTES that were induced with a more delayed kinetic, peaking within 10–24 h p.i. Expression of IFN- mRNA was gradual, peaking by 10 h and remaining high at 48 h p.i., whereas expression of the IFN-inducible genes, Mx-1 and Mx-2, were consistently induced following the peak of IFN-β, peaking by 24 h and remaining detectable until 96 h p.i. (Fig 2). In addition, VANBT infection induced the proinflammatory cytokines interleukin-6 (IL-6) and IFN-γ with different peak times. IL-6 peaked at 4 h, while IFN-γ showed a late expression, remaining higher at 24–48 h p.i. (Fig 2). We additionally measured the expression of Th2 cytokines (*e*.*g*., IL-4, IL-5, IL-13, and IL-10), but none of them showed significant expression in the lung tissues upon VANBT infection (see below).

**Fig 3:**
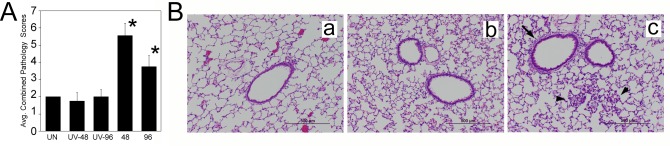
VANBT infection in cotton rats causes lung pathology. (A) Histopathology scores obtained from lungs of uninfected (UN) or UV-VANBT or VANBT infected animals and euthanized at the indicated time p.i. Combined scores represent extent of peribronchiolitis, perivasculitis, interstitial inflammation and alveolitis. n = 4–8, *p<0.05 for VANBT compared to UV-VANBT at respective time point. (B) Representative H&E-stained lung sections in naïve rats (a) or lungs taken after 48 h after i.n. inoculation with UV-VANBT (b), or VANBT (c). Peribronchial inflammation (black arrow) and alveolitis (black arrow head) are indicated. Scale bars, 500 μm. Images shown are representative of four cotton rats.

Consistent with the results of pulmonary inflammatory gene expression, lung pathology was moderate and peaked at 48 h p.i., and was characterized by the presence of a mild, but significant, increase in cumulative pathology that included bronchiolitis, perivasculitis, interstitial pneumonia, and alveolitis (Fig 3A). Examination of H&E-stained lung sections revealed areas of extensive peribronchial and alveolar cellular infiltration in VANBT-infected animals, which were not evidenced in the lungs of control UV-VANBT inoculated animals (Fig 3B).

### Intramuscular immunization with EV-D68 strains enhances NA response

**Fig 4:**
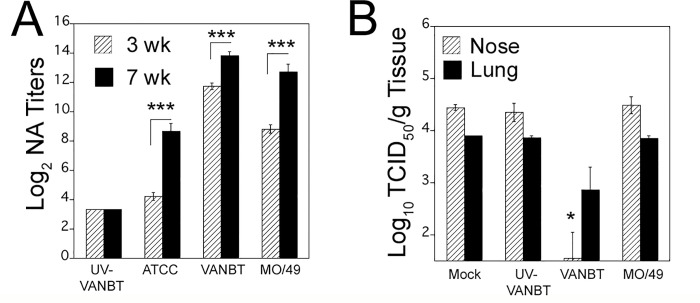
Intramuscular immunization with live EV-D68 strains induces virus-specific homologous NA titers. (A) Female cotton rats were immunized i.m. with 10^6^ TCID_50_/100 μl of indicated virus on day 0 and boosted 3 weeks after the first immunization. Serum samples were obtained at 3 week (before boosting) and 7 weeks after the first immunization and homologous serum NA titers were determined. The sera were assayed in duplicate and NA titer is expressed as Log_2_ geometric mean ± SE. n = 10–15 from two independent experiments, ***p< 0.001 for 3 weeks NA titer compared with 7 weeks NA. (B) Passive transfer of VANBT immune sera protects animals from VANBT challenge. Animals were treated intraperitoneally with 0.5 ml 1 x PBS (mock) or serum from animals immunized i.m. with either UV-VANBT, VANBT, or MO/49. The following day, animals were challenged i.n. with 10^6^ TCID_50_ of VANBT and euthanized 10 h later to determine nose and lung viral titers. n = 4–5 per group. * p<0.05 for each group compared with mock group.

As NA are pivotal for prevention of viral infection or blocking viral replication, we next investigated the effect of i.m. immunization with live EV-D68 strains on generation of humoral protective immune responses by measuring serum NA against different EV-D68 strains. We immunized groups of adult cotton rats i.m. with each of the three EV-D68 strains, either live or UV-inactivated, at the dose of 10^6^ TCID_50_ at day 0 and boosted 3 weeks after the first immunization with the same dose of virus. Serum samples were collected from animals at 3 (before boosting) and 7 weeks after the first immunization and homologous and heterologous NA levels were determined by *in vitro* neutralization assay. No signs of clinical disease were seen in animals immunized i.m. with either inactivated or live viruses. Immunization with live ATCC, VANBT, or MO/49 induced homologous NA titers in sera at 3 weeks post-immunization, which were further increased at 7 weeks due to boosting (Fig 4A). At 7 weeks, serum samples from animals immunized with live ATCC showed the lowest NA titer (log_2_ 8.7±0.5), followed by the group immunized with MO/49 (log_2_ 12.7±0.5), whereas animals immunized with live VANBT showed the highest titer (log_2_ 13.8±0.3) (Fig 4A and Table 1). The intensity of induction of homologous NA titer followed i.m. immunization with live virus mirrors their respective infectivity in cotton rats (Fig 1B and 1C). Similar to naïve animals, i.m. immunization with UV-VANBT did not induce detectable NA titer (Fig 4A, Table 1). Next, we tested whether VANBT replicates at the site after i.m. inoculation and thus enhance NA production. Groups of cotton rats were mock-inoculated i.m. with 1x PBS, or inoculated i.m. with live VANBT and sacrificed at 2, 5, and 15 h post inoculation. VANBT (-) vRNA was measured in RNA samples extracted from inguinal and lumbar lymph nodes, draining from the site of inoculation. (-) vRNA strands were detected in lymph nodes of VANBT inoculated animals (S4 Fig). The levels of (-) vRNA at 2 and 5 h were almost comparable, and decreased by 15 h post inoculation.

**Table 1:** Homologous and Heterologous serum NA titer in EV-D68 immunized cotton rats.

Immunogen^a^	n^b^	NA^c^		
		ATCC	VANBT	MO/49
PBS	5	3.3 ± 0.0^d^	3.3 ± 0.0	3.3 ± 0.0
UV-VANBT	15	3.3 ± 0.0	3.3 ± 0.0	3.3 ± 0.0
ATCC	15	8.7 ± 0.5	3.5 ± 0.1	3.3 ± 0.0
VANBT	15	8.6 ± 0.6	13.8 ± 0.3	7.2 ± 0.5
MO/49	10	5.1 ± 0.8	6.9 ± 0.8	12.7 ± 0.5

^a^Female cotton rats were immunized i.m. with 10^6^ TCID_50_/100 μl of indicated virus on day 0 and at 3 weeks. Serum was obtained at 7 weeks.

^b^Number of cotton rats used in each vaccination group.

^c^Geomean ± SE of Log_2_ NA titers. Highlighted values represent homologous titers.

^d^Limit of detection of the assay.

Next, we sought to determine the capacity of the EV-D68-immune serum samples to neutralize viruses of different clades used for immunization. For testing cross-reactivity, an immune serum against one virus strain was used to measure its ability to neutralize the other two virus strains *in vitro*. As shown in Table 1, ATCC immune sera generated measurable homologous NA titers, but negligible NA titers against VANBT and MO/49. Both VANBT and MO/49 immune sera showed strong homologous NA responses and also moderate heterologous response against the other two strains. However, VANBT appeared to be the strongest inducing NA responses against all the three strains (Table 1). Serum samples from mock (PBS) or from animals immunized with UV-VANBT showed no neutralizing activity against all the EV-D68 strains. These data strongly suggest that immunization with VANBT substantially induces a broader NA response that could be protective against heterologous EV-D68 challenge.

As VANBT and MO/49 both generated a strong and a moderate NA response, respectively, against all three viruses, we next tested the efficacy of the prophylactic administration of serum from EV-D68 immunized (VANBT and MO/49), UV-VANBT immunized, or mock treated animals to protect against i.n. VANBT challenge. All animals that received immune sera with high NA antibodies against VANBT intraperitoneally prior to challenge (average NA titer of VANBT immune sera = 13.7 Log_2_) showed a significant reduction in the nose and the lung viral titers by ~1 Log_10_ at 10 h p.i., whereas animals that were mock-treated or that received UV-VANBT or MO/49 sera prophylactically, showed no significant protection (Fig 4B). These data demonstrate that passive transfer of antibodies can be considered an effective prophylactic therapy against EV-D68 infection.

### Intramuscular immunization with EV-D68 accelerates virus clearance

**Fig 5:**
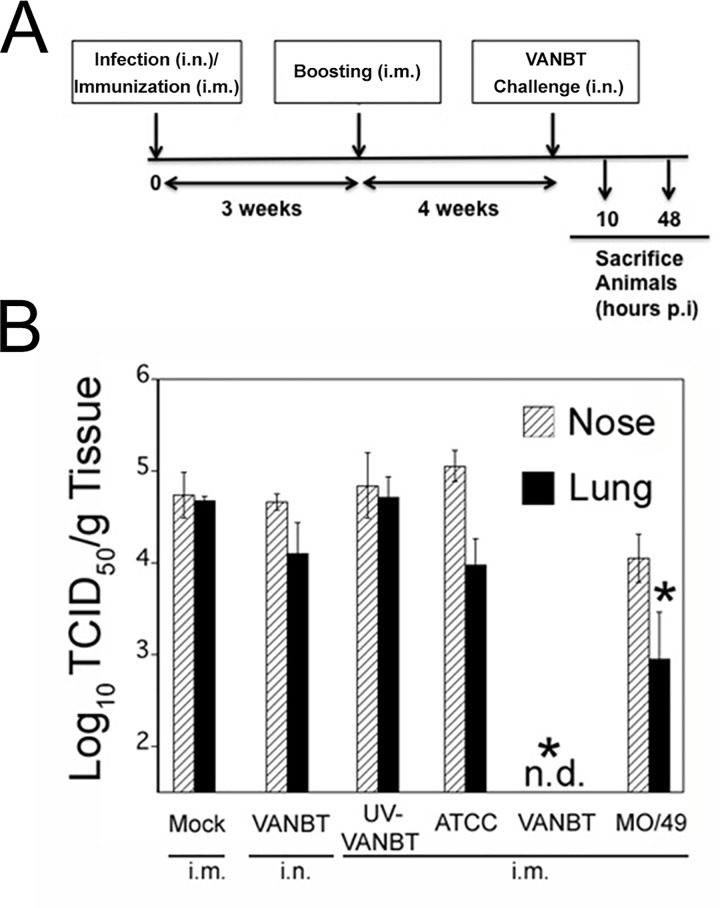
Intramuscular immunization with EV-D68 protects against VANBT challenge. (A) Schematic representation of infection and immunization regimen. Female cotton rats were mock-immunized i.m. on day 0 and at 3 weeks, infected with VANBT on day 0, or immunized i.m. on day 0 and at 3 weeks with UV-VANBT, ATCC, VANBT, or MO/49 using 10^6^ TCID_50_/100 μl of virus. At 7 weeks, all animals were challenged i.n. with 10^6^ TCID_50_ of VANBT and sacrificed at 10 h or 48 h p.i. (B) Viral titers in the nose and lung of animals sacrificed at 10 h p.i. n = 5 per group. Data are representative of two independent experiments. * *p*<0.05 where each group is compared with mock-immunized group.

We tested whether the NA responses induced by EV-D68 immunization were sufficient to confer protection against the virus challenge. Groups of cotton rats were mock immunized i.m., infected with VANBT i.n., or immunized i.m. with UV-VANBT, or live ATCC, live VANBT, or live MO/49 (Fig 5A). All animals were immunized on day 0 and boosted at 3 weeks, whereas the VANBT-infected group was inoculated on day 0. At week 7, all animals were challenged i.n. with VANBT and sacrificed at 10 h p.i. to measure nose and lung viral load. As shown in Fig 5B, i.m. immunization with live VANBT resulted in complete and rapid virus clearance in both nose and lung tissues. No protection was detected in mock-immunized animals, animals previously infected with VANBT, animals immunized with UV-VANBT, or animals immunized with live ATCC (Fig 5B). However, MO/49-immunized animals showed a moderate reduction in viral titer in nose and lung tissues by ~0.7 Log_10_ and ~1.7 Log_10_, respectively (Fig 5B), correlating with the generation of the cross-protective NA titers in this model (Table 1).

**Fig 6:**
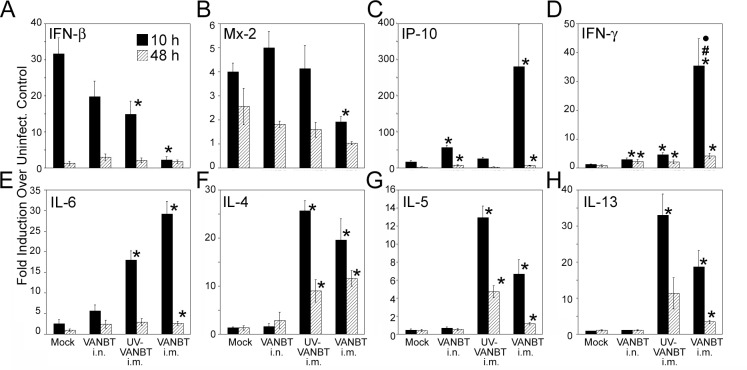
Intramuscular immunization with VANBT induces both Th1 and Th2 cytokines upon virus challenge. Female cotton rats were mock-immunized i.m. on day 0 and at 3 weeks, infected with VANBT on day 0, or immunized i.m. on day 0 and at 3 weeks with UV-VANBT, or VANBT, using 10^6^ TCID_50_/100 μl of virus. At 7 weeks, all animals were challenged i.n. with 10^6^ TCID_50_ of VANBT and sacrificed at 10 h or 48 h p.i. Relative mRNA expression profiles of IFN-β (A), Mx-2 (B), IP-10 (C), IFN-γ (D), IL-6 (E), IL-4 (F), IL-5 (G), and IL-13 (H) at either 10 or 48 h p.i. in the lung tissues were measured by qRT-PCR. Results were calculated as fold-induction over uninfected (naïve) animals and expressed as geometric means ± SE. Results are representative of two independent experiments, n = 5. * p<0.05 for each group compared with mock-immunized group, #p<0.05 when VANBT/i.m. compared to VANBT/i.n. and ⦁p<0.05 when VANBT/i.m. compared with UV-VANBT/i.m.

Next, we examined the effect of VANBT i.m. immunization on the type and kinetics of pulmonary cytokines and IFN responses after VANBT challenge. We analyzed animals mock-immunized i.m. (as primary infection), previously infected with VANBT i.n. (as secondary infection), or immunized i.m. with either UV-VANBT or live VANBT. As shown for primary infection, VANBT induced strong IFN-β mRNA expression, peaking at 10 h p.i. in the lungs (Fig 2). We measured IFN-β and Mx-2 expression at 10 and 48 h p.i. in the immunized groups (Fig 6A and 6B). Immunization with live VANBT significantly reduced expression of IFN-β and Mx-2 at 10 h p.i. compared to mock-immunized group, which is consistent with complete protection seen in this group (Fig 5B). In addition, animals previously infected with VANBT i.n. or immunized with UV-VANBT i.m. showed decreased expression of IFN-β, but not of Mx2 (Fig 6A and 6B). Moreover, immunization with live VANBT showed a dramatic increase in the expression of chemoattractant IP-10 at 10 h p.i., which remained elevated even at 48 h p.i. (Fig 6C). Immunization with live VANBT elicited a strong Th1 (IFN-γ) cytokine response in the lung tissues at 10 h, which remained even higher at 48 h p.i. (Fig 6D). Animals previously infected with VANBT i.n. or immunized i.m. with UV-VANBT showed higher expression of IFN-γ compared to mock-immunized group, although the magnitude of IFN-γ induction in live VANBT-immunized group was higher than these two groups (#p<0.05 when VANBT/i.m. compared to VANBT/i.n. and ⦁p<0.05 when VANBT/i.m. compared with UV-VANBT/i.m. at 10 h p.i.). Furthermore, both UV-VANBT and VANBT-immunized groups showed significant induction of signature Th2 cytokines IL-6, IL-4, IL-5, and IL-13 mRNA expression at both 10 and 48 h p.i. (Fig 6E–6H).

**Fig 7:**
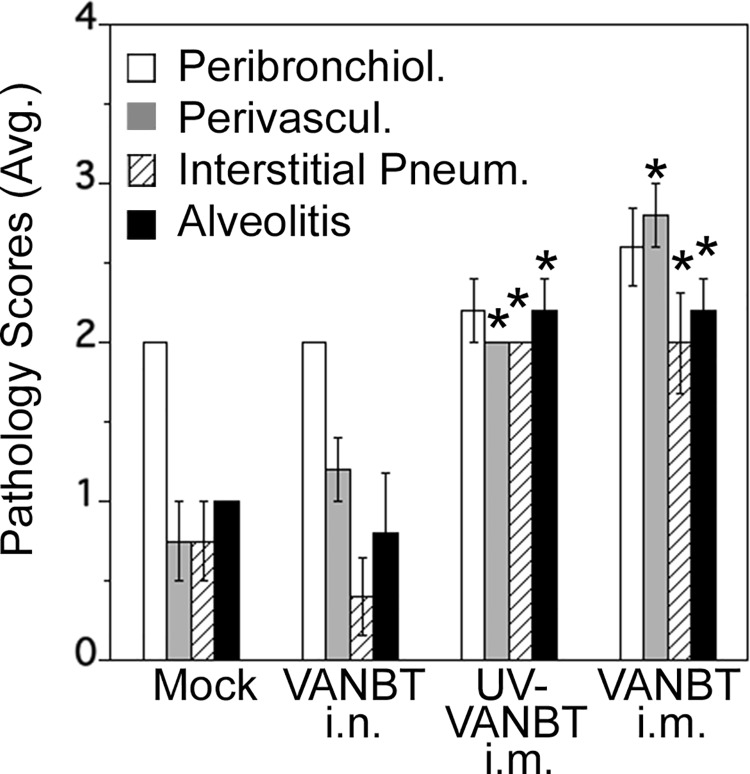
Lung histopathology scores of VANBT-immunized cotton rats upon virus challenge. Groups of female cotton rats were infected or immunized as described above for Fig 6. At 7 weeks, all groups were challenged with VANBT i.n. and sacrificed at 48 h p.i. Graphs represent the average scores for bronchiolitis, vasculitis, interstitial pneumonia, and alveolitis. Results are representative of two independent experiments, n = 5. * p<0.05 for each group compared with mock immunized group or VANBT/i.n. group.

We determined the extent of lung pathology in all the four groups by scoring H&E-stained lung sections. As shown in Fig 7, immunization with both UV-VANBT and live VANBT i.m. showed significantly increased scores for vasculitis, interstitial pneumonia and alveolitis, compared to mock-immunized, or the previously infected with VANBT i.n. group, which suggests an exacerbated inflammatory response in these groups.

## Discussion

In the past decade, the detection frequency of EV-D68 in respiratory infections has been on the rise worldwide and the 2014 USA outbreak was the largest and most widespread EV-D68 epidemic investigated to date [9, 10, 13–23]. Increasing numbers of EV-D68 cases showing acute flaccid paralysis and cranial nerve dysfunction in children raised concerns about its potential impact on public health [27–29, 41, 42]. However, the underlying causes for this upsurge of EV-D68 infections are still unknown. Association of different viral subclades/lineages and disease severity has been described, but recent reports have found contradictory results [31, 41, 43].

EV-D68 strains circulating in recent years have been divided into three distinct clades A, B, and C based on VP1 phylogenetic tree [9, 10]. Since the first isolation of the virus, mutations have accumulated in the BC and DE loops of the VP1 sequence, which corresponds to region of the protein on the viral surface that are associated with antigenic epitopes, indicating that unique sequence variations in these loops may cause a shift in the antigenicity [19, 22, 43]. In the current study, we used three different strains, (1) classical ATCC (prototype Fermon strain) that was used as a reference strain before the more abundant circulation of the virus [8, 33]; (2) pre-outbreak isolate VANBT collected in 2012 that belongs to subclade A1, which is considered to be endemic and isolated in many different geographical regions [13, 15, 16, 18–22]; and (3) outbreak isolate MO/49, collected in 2014 that belongs to subclade B1, which is believed to be dominant during the 2014 USA outbreak [31, 32, 41]. Analysis of these 3 strains in the cotton rat model has led to our conclusion that their replication and immunological profiles differ significantly.

It has been reported that EV-D68 binds to α2,6-linked SAs; however, the lack of a suitable animal model has severely limited the study of EV-D68 infection, immunity, and pathogenesis to test methods of intervention and prevention strategies [33, 34]. The species of the cotton rat (genus *Sigmodon*) has been shown to be a preferred model for an impressive list of human pathogens including respiratory syncytial virus (RSV), influenza (A and B serotypes), adenoviruses (several serotypes), parainfluenza virus (type 3), measles, herpes simplex (types 1 and 2), and human metapneumovirus [35, 36, 44–48]. For many of these viruses, the cotton rat represents a reliable model that better mimics human disease [49]. In fact, picornaviruses were the first group of viruses studied in cotton rats. Initial studies of poliovirus demonstrated that the Lansing strain of poliomyelitis was selected for replication in mice by a process of adaptation involving serial passage of viruses in cotton rats [50]. Moreover, It was shown that EV-A71 strain, isolated from samples of the brain and feces of children during an outbreak in Bulgaria in 1975, induced a paralytic disease in newborn and adult cotton rats [51]. We recently showed that cotton rats are permissive to infection with HRVs, and i.n. infection resulted in recovery of infectious viral load in the lung until 2 days p.i., accompanied by significant pathology, mucus production, and expression of inflammatory mediators [37].

As there are no current animal models for EV-D68 and information regarding relevant disease-related outcomes from human cases is limited, we herein characterized EV-D68 infection in cotton rats by measuring viral load and vRNA (negative or total) in either nose or lung tissues until 4 days p.i., assessing mRNA expression of various chemokines, IFNs, and proinflammatory cytokines, and lung histology. We focused on analysis of the nose and lung tissues since most recent cases of EV-D68 showed the respiratory tract as the most evident target for infection with this virus [10, 15, 22]. Our data clearly shows that cotton rats are permissive to EV-D68 and that it replicates in respiratory airways, although with differing infection profiles among three strains, ATCC being the weakest, while VANBT showed the strongest infection and replication profile (Fig 1B). These differences in viral replication could represent different evolutionary stages of the three strains that can affect at various levels of replication of these viruses or they may differentially use an alternative, nonsialylated receptor to infect the cells [52]. Although the replication cycle of VANBT appeared to be short-lived, its profile of viral titer in the nose over time (showing clear virus eclipse at 4 h, rising quickly and reaching a peak by 10 h p.i.), and their differences with the output virus obtained after infection with different strains demonstrate that VANBT replicates in upper respiratory tract of cotton rats. Replication of VANBT was studied further by measuring viral replication intermediate, (-) vRNA, which showed early production (showing a peak at 4 h p.i.) of replicative RNA in the lungs. As viral genome replicating intermediates, (-) vRNA showed peak expression prior to detection of peak infectious virus titer, it confirms that we are detecting early products of viral replication, which eventually increased the viral titer at ~10 h p.i. Since we could detect VANBT primarily in the nose and lung tissues and infectious virus decreased after 10 h p.i., it is possible that VANBT could infect only a limited amount or type of cells in the respiratory tract of cotton rat with reduced transmission to other tissues. The 2014 USA outbreak of acute flaccid paralysis occurred at the same time as a far larger outbreak of EV-D68 across the USA. The vast majority of confirmed cases of EV-D68 mostly showed development of only respiratory illness and viral presence was confirmed in only a handful of the paralyzed children, which emphasize that the EV-D68 tropism is primarily directed to the upper and lower respiratory tract [11, 53]. Although we do not rule out potential changes in tropism in our model, these events could be, as in humans, very rare and need further investigation.

Our data shows that VANBT infection causes temporally defined expression of lung cytokines and elicits lower respiratory tract pathology in cotton rats. The absence of pathologic, inflammatory, or antiviral responses (induction of IFN-β and IFN-inducible genes Mx-1 and Mx-2) in rats inoculated with UV-VANBT indicates that these responses were replication-dependent. Mx proteins possess GTPase activity and the ability to form oligomers, which are important to mediate IFN-dependent antiviral activity [54]. Induction of timely antiviral responses in cotton rats by fully functional Mx genes may underlie the short-lived replication cycle of VANBT in cotton rats. As we observed mild inflammation in the lung of infected animals, there is a possibility that only limited cells could be infected by VANBT in cotton rats. However, it has been suggested that healthy adults usually show a milder range of respiratory symptoms upon EV-D68 infection, similar to that described here in cotton rats [18, 53, 55]. Interestingly, EV-D68 infection was reported in adult patients with hematologic malignancy and hematopoietic cell transplant recipients, showing mild upper respiratory symptoms to respiratory failure [56]. Thus, it would be important to measure VANBT replication in immunocompromised (cyclophosphamide treatment induced) cotton rats or blocking IFN-β production in cotton rat upper respiratory tract cells *in vitro* [57]. As children are at higher risk for severe respiratory symptoms due to EV-D68 infection, it would be pertinent to evaluate EV-D68 replication in younger cotton rats or cotton rat pups that are only a few days old [53, 58].

In a tight correlation with the significant differences in the infection profile by three strains of EV-D68 in cotton rats, we observed that the strains differ in their capacity to induce NA followed by i.n. infection or i.m. immunization. ATCC induced the lowest titers, while VANBT elicited the highest homologous NA response (Fig 1F, Fig 4A and Table 1). On the basis of hemagglutination inhibition (HI) and NA titers, it was previously shown that the Fermon strain had lower HI and NA titers than recently detected EV-D68 strains, which may explain that ATCC has lower antigenicity leading to induction of lower NA titers. The HI and NA titers were also shown to be significantly different between strains of different genetic clades among recently detected EV-D68 strains [33]. Compared to VANBT and MO/49, ATCC elicited essentially negligible heterologous NA titers (Table 1). A recent study using the Bayesian Markov chain Monte Carlo approach have estimated that genetic diversity in the VP1 region increased after the late 1990s, which may have resulted in the emergence of the three clades [22]. This suggests that ATCC, being the prototype strain, and isolated in 1962, could differ vastly in terms of its antigenic characteristics, and even replication in humans, and as shown here, in cotton rats, when compared with the more recent VANBT and MO/49 strains.

Previously, we showed that i.n. infection of cotton rats with HRV1B or HRV16 at comparable doses, only HRV16 showed high levels of virus replication in nose, trachea, and lungs, whereas HRV1B showed significantly lower infectious virus titers in these tissues [37]. Furthermore, we reported that i.m. live HRV16 vaccination of cotton rats generated high titers of NA in all vaccinated animals, while HRV1B did not elicit detectable homologous NA [37]. With HRVs, we have found a strong correlation between production of high titers of serum NA against certain HRVs and the ability of these HRVs to replicate in the airways of cotton rats. As there were vast differences among the three strains of EV-D68 in infectivity profile and NA titer generation followed by infection, we think that it correlate to RV infection in cotton rats. Our data showed that VANBT replicates in the lymph nodes draining from the site of i.m. inoculation (S4 Fig), thus increasing the chance of antigen processing and presentation by macrophages and dendritic cells to initiate the robust immune response.

Our results with passive transfer of EV-D68 immune sera showed that prophylactic administration of VANBT hyper-immune serum protects both nose and lung tissues of naïve animals against VANBT challenge, indicating that passive antibody transfer could be a potentially effective prophylactic therapy against EV-D68 infection during outbreaks or for treatment of individuals at high-risk (Fig 4B). Prophylactic antibody therapy for infectious diseases has become an important alternative in the absence of a vaccine, such as RespiGam^TM^ and Synagis^TM^ for RSV and recently ZMapp^TM^ for Ebola virus [59–61].

We further assessed different immunization using live and UV-inactivated EV-D68 against VANBT challenge. We found that i.m. immunization with live VANBT and MO/49, but not live ATCC, resulted in a decrease of viral loads in nose and lung tissues (Fig 5B). Though i.n. infection with VANBT resulted in induction of NA titer (Fig 1G), animals previously infected with VANBT did not show any significant protection against re-infection (Fig 5B), which suggests that infection with EV-D68 fails to provide the degree of immunity induced by infection with other respiratory viruses in this model, *i*.*e*., influenza and RSV, where infection induces complete protection to re-infection [62–65]. However, similar results were seen for i.n. infection with HRV that replicate in cotton rats, but does not protect against re-infection [37]. The moderate protection achieved by live MO/49 immunization against VANBT challenge (Fig 5B) is consistent with its capacity to generate detectable heterologous NA against VANBT (Table 1). Our results clearly demonstrate generation of cross-strain NA response conferred by immunization with live EV-D68 belonging to different clades, which could serve as a tool to define conserved antigenic features to use as candidates to induce broad-spectrum immunity against different clades of EV-D68 and thus warrants further investigation. On the basis of pulmonary cytokine analysis on EV-D68 immunized animals after challenge, we conclude that live i.m. VANBT-immunized animals mount both strong Th1 and Th2 responses and UV-VANBT i.m. immunized group showed excessive Th2 response that could predispose to the development of allergic responses. Similarly, it was previously shown that UV-inactivated viruses used for immunization have the propensity to develop Th2-biased responses and enhanced respiratory disease [66, 67]. For the first time, this study assessed the effect of immunization with crude vaccine preparations for EV-D68 given i.m. Despite the efficacy of i.m. immunization with VANBT to reduce viral titers in vivo, animals vaccinated i.m. with UV-VANBT and VANBT enhanced cytokine responses and lung histopathology, which raises the possibility that pre-existent immunity to these viruses could have deleterious effects. Association of EV-D68 with acute flaccid paralysis in children definitively raises safety issues for vaccine developers. Our data serve to emphasize that a live EV-D68 vaccine generates high titer NA and could be useful to define mechanisms of immunity against the virus. Future studies to develop either live-attenuated vaccine, inactivated EV-D68 whole virus vaccine, virus-like particle-based vaccines, recombinant VP1 protein based vaccines with varied dosage, or inclusion of adjuvants to formulate safe vaccines for EV-D68 are logical next steps to reduce potential adverse effects. In this regard, we have previously shown that a formalin-inactivated RSV vaccine, shown to induce “vaccine enhanced disease” in clinical trials in the mid-1960’s, could be rendered safe in this model by inclusion of the TLR4 agonist adjuvant, monophosphoryl lipid A [68].

We used EV-D68 clinical isolates from human patients without any adaptation to infect cotton rats. This is one of the biggest advantages of our model because it is clear that virus adaptation introduces a considerable number of nucleotide variations in viral genome that may dramatically alter the course of natural infection [35, 69]. Despite the variety of geographical sources for EV-D68, the strains detected in recent years have similar VP1 sequences as long as they belong to the same genetic clade [33]. Previous studies have shown that viruses of the clades A to C circulated or co-circulated during different time periods in different geographic locations [9, 10, 14–17, 19, 21, 22]. Thus, the possibility of antigenic differences of strains belonging to same clade/subclade but different geographical distance is minimal [18]. Therefore, we hypothesize that the cotton rat model could be an important tool to infect other current or future EV-D68 isolates detected in other geographical locations to assess vaccine strategies and antiviral molecules against EV-D68.

## Materials and Methods

### Ethics statement

All animal work presented in this paper was conducted under strict accordance with the recommendations in the Guide for the Care and Use of Laboratory Animals of the National Institutes of Health. The animal protocols (protocol # 2 and 7) were approved by the Institutional Animal Care and Use Committee (IACUC) of Sigmovir Biosystems Inc. (SBI) (OLAW assurance #A4642-01). SBI has USDA breeding and Research licenses (51-A-0031 and 51-R0091, respectively), and is accredited by the Association for Assessment and Accreditation of Laboratory Animal Care (AAALAC).

### Animals

Four to six week old female cotton rats were obtained from the inbred colony maintained at SBI. Cotton rats in the colony were seronegative for EV-D68 by neutralization assay, and seronegative by ELISA to other adventitious respiratory viruses (*e*.*g*., Pneumonia virus of mice, rat parvovirus, rat coronavirus, Sendai virus). Animals were housed in large polycarbonate cages, and fed a diet of standard rodent chow and water *ad libitum*. Cotton rats were infected i.n. or immunized intramuscularly (i.m.) with three different strains of EV-D68 under isoflurane anesthesia by inoculation of 100 μl of virus preparation [10^6^ 50% Tissue Culture Infective Dose (TCID_50_)] per rat. Serum samples were obtained by retro-orbital blood collection under isoflurane anesthesia. Animals were euthanized by carbon dioxide asphyxiation.

### Viruses

Three strains of EV-D68 were studied: (1) ATCC (Accession # KT725431), which is the prototype Fermon virus strain purchased from American Type Culture Collection (ATCC, Manassas, VA), (2) VANBT/1 (supplied by Dr. Tina V. Hartert; Accession # KT347280) collected in 2012 from Nashville, TN at Vanderbilt University Medical Center as a pre-outbreak isolate, and (3) MO/14/49 (BEI resources, NIAID; Accession # KM851227) collected in Kansas city during the 2014 outbreak in USA [8, 31, 32] (Fig 1A). Virus stocks were produced in HeLa H1 cells (ATCC, Manassas, VA) grown in DMEM (Lonza) with 10% FBS (Seradigm) at 34°C and 5% CO_2_. Five days p.i., cells were freeze-thawed 4–5 times and vortexed vigorously to release the cell associated viruses and centrifuged at 800 x g before determination of TCID_50_. Replication-deficient virus was generated by exposing the virus stocks to ultraviolet light for 30 min.

### Virus titration assay and Neutralization antibody assay

Tissue samples (left lung lobe and entire nose) were homogenized in 3 ml of homogenization buffer (EMEM, 1X SPG, 1% Fungizone, 0.1% Gentamycin) at different time-points p.i. Infectious virus titers were determined by standard endpoint dilution assay and expressed as TCID_50_/g of tissue. Briefly, after overnight incubation, HeLa H1 cells in 96 well plates were washed with serum free medium and infected with serial 10-fold diluted freeze-thawed tissue homogenates and cell viability was assessed by crystal violet staining. Each assay was repeated at least three times with three to five replicates per assay. Virus neutralization was performed by incubation of serial 2-fold dilution of immunized and control cotton rat sera with 200 TCID_50_ virus/well in 96 well plate. After 5 days of incubation at 34°C, cell viability was assessed. Each assay was repeated at least three times with four replicates per assay. Each run contained cell control plate and back titration plate for each EV-D68 virus.

### RNA isolation and qRT-PCR analysis

RNA was isolated from the lung lingular lobe using the RNAeasy kit (Qiagen Sciences). cDNA was prepared by QuantiTect Reverse transcription kit (Qiagen Sciences) or SuperScript® II Reverse transcriptase (Thermo Fisher Scientific). Each cDNA reaction was prepared from 1 μg of initial RNA and diluted to 100 μl of the final volume. Three μl of cDNA was subsequently used for each PCR reaction. EV-D68 specific qRT-PCR was developed using primers that target the VP1 of EV-D68 (S1 Table). To quantify viral replication, cDNA for the negative strand viral RNA (as an indicator of active RNA transcription) was synthesized by priming with a forward primer flanking at the start of the VP1 sequence (EVD68 VP1 F1/1979 primer). The PCR reaction was performed using internal nested primers (VANBT VP1 F3/1983 and VANBT VP1 R3/1984). The entire VP1 PCR amplicon (using primers EVD68 VP1 F1/1979 and EVD68 VP1 R1/1980) was gel purified and diluted to generate a copy number standard curve, which was used to quantify copies/g tissue of negative viral RNA. The assessment of cotton rat cytokines mRNA expression was carried out using primers as previously described [70, 71].

### Lung Histopathology

Lungs were dissected *en bloc* and right lobe was inflated with 10% neutral buffered formalin to their normal volume, and submersed in the same fixative solution. Following fixation, lungs were embedded in paraffin, sectioned, and stained using hematoxylin and eosin (H&E). Four parameters of pulmonary inflammation were evaluated: peribronchiolitis (inflammatory cell infiltration around the bronchioles), perivasculitis (inflammatory cell infiltration around the small blood vessels), interstitial pneumonia (inflammatory cell infiltration and thickening of alveolar walls), and alveolitis (cells within the alveolar spaces). Slides were scored blindly on a 0 to 4-severity scale (absent, minimal, mild, moderate and marked).

### Statistical Analysis

Viral titers, NA titers, expression of cytokine genes and total vRNA or (-) vRNA were calculated as geometric means ± standard error (SE) for all animals in a group at a given time p.i. Student *t*-test was used to determine statistically significant differences between two groups, using an unpaired, two-tailed test with significance set at *p*<0.05. Pulmonary pathology scores were expressed as the arithmetic means ± SE for all animals in a group.

## Supporting Information

S1 Fig Comparison of VANBT replication in male and female cotton rats. Groups of 5 male and female cotton rats of matched ages were infected i.n. with 10^6^ TCID_50_ of VANBT and euthanized at 10 h p.i. Nose and lung viral titers were compared between the two groups.
(TIF)

S2 Fig Time course of VANBT replication in cotton rats. Groups of 4 cotton rats were infected i.n. with 10^6^ TCID_50_ of VANBT, euthanized at the indicated time p.i. and total vRNA was quantified by qRT-PCR in lung tissue. Animals inoculated with UV-VANBT and sacrificed at 6 h p.i. (UV-6) were shown as control.
(TIF)

S3 Fig Persistence of homologous NA titer followed by i.n. VANBT infection. Group of 10 cotton rats were infected i.n. with 10^6^ TCID_50_ of VANBT and serum was obtained at 3 weeks and 9 weeks p.i. Homologous serum NA titers were determined using in vitro neutralization assay.
(TIF)

S4 Fig Replication of VANBT in the lymph nodes surrounding site of i.m. inoculation. Groups of 3 cotton rats were inoculated i.m. with live 10^6^ TCID_50_ of VANBT and sacrificed at 2, 5, and 15 h post-inoculation (n = 3/per time point). Two animals inoculated with 1x PBS and sacrificed at 15 h (Mock-15) post-inoculation were shown as control. Inguinal and lumbar lymph nodes, near to the site of injection, were collected from each animal at the indicated time point and (-) vRNA was quantified by qRT-PCR.
(TIF)

S1 Table Nucleotide sequence of primers used for either reverse transcription or quantitative PCR for detecting vRNA. (TIF)
