# Supplementary figures and images for: Enterovirus D-68 Infection, Prophylaxis, and Vaccination in a Novel Permissive Animal Model, the Cotton Rat (Sigmodon hispidus)
Source: PLoS One. 2016 Nov 4;11(11):e0166336. doi: 10.1371/journal.pone.0166336 (PMC5096705; doi:10.1371/journal.pone.0166336)

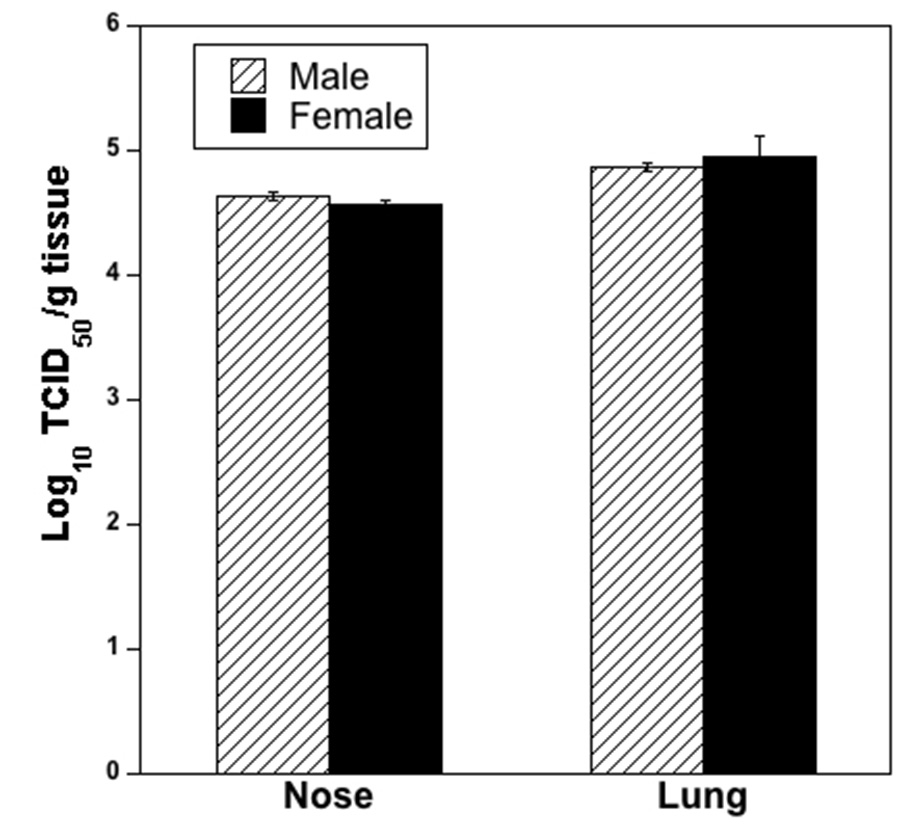

Supplement: S1 Fig — Groups of 5 male and female cotton rats of matched ages were infected i.n. with 106 TCID50 of VANBT and euthanized at 10 h p.i. Nose and lung viral titers were compared between the two groups. (TIF) [file pone.0166336.s001.tif]

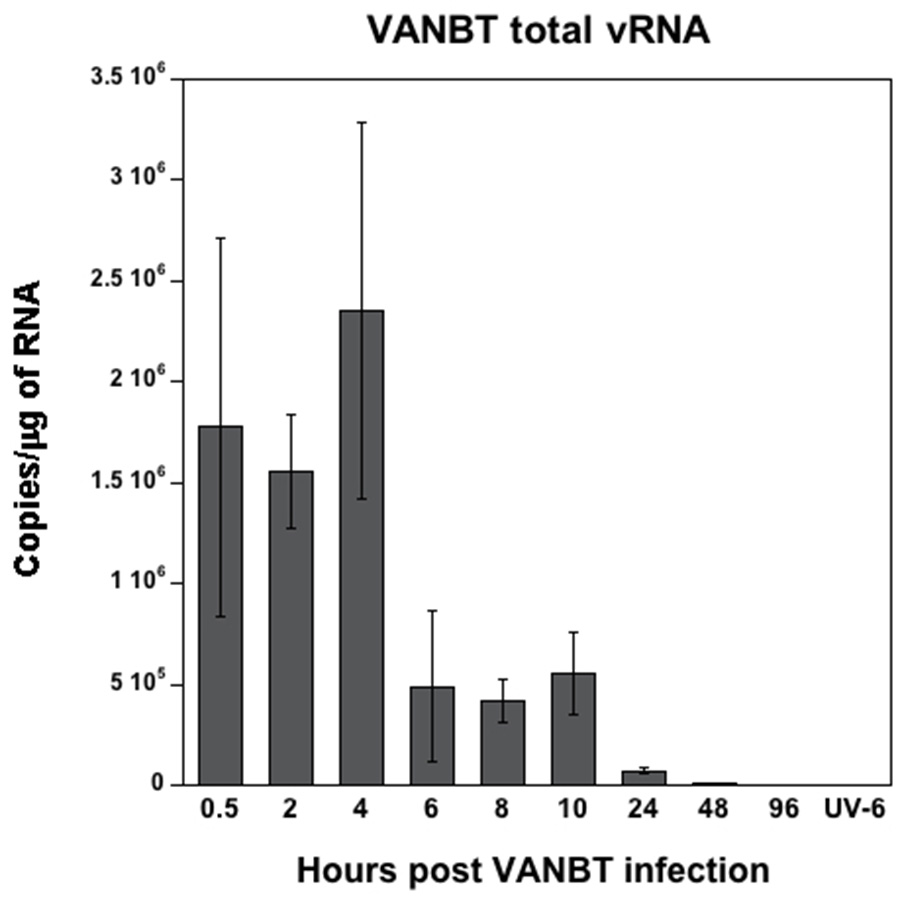

Supplement: S2 Fig — Groups of 4 cotton rats were infected i.n. with 106 TCID50 of VANBT, euthanized at the indicated time p.i. and total vRNA was quantified by qRT-PCR in lung tissue. Animals inoculated with UV-VANBT and sacrificed at 6 h p.i. (UV-6) were shown as control. (TIF) [file pone.0166336.s002.tif]

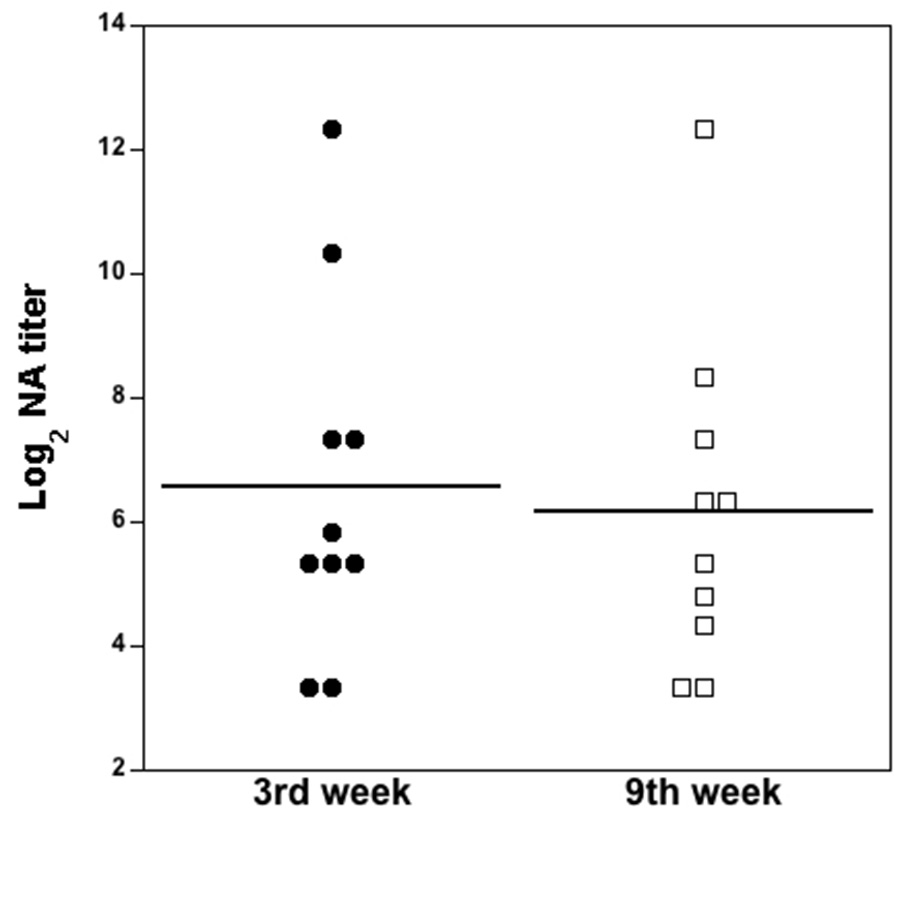

Supplement: S3 Fig — Group of 10 cotton rats were infected i.n. with 106 TCID50 of VANBT and serum was obtained at 3 weeks and 9 weeks p.i. Homologous serum NA titers were determined using in vitro neutralization assay. (TIF) [file pone.0166336.s003.tif]

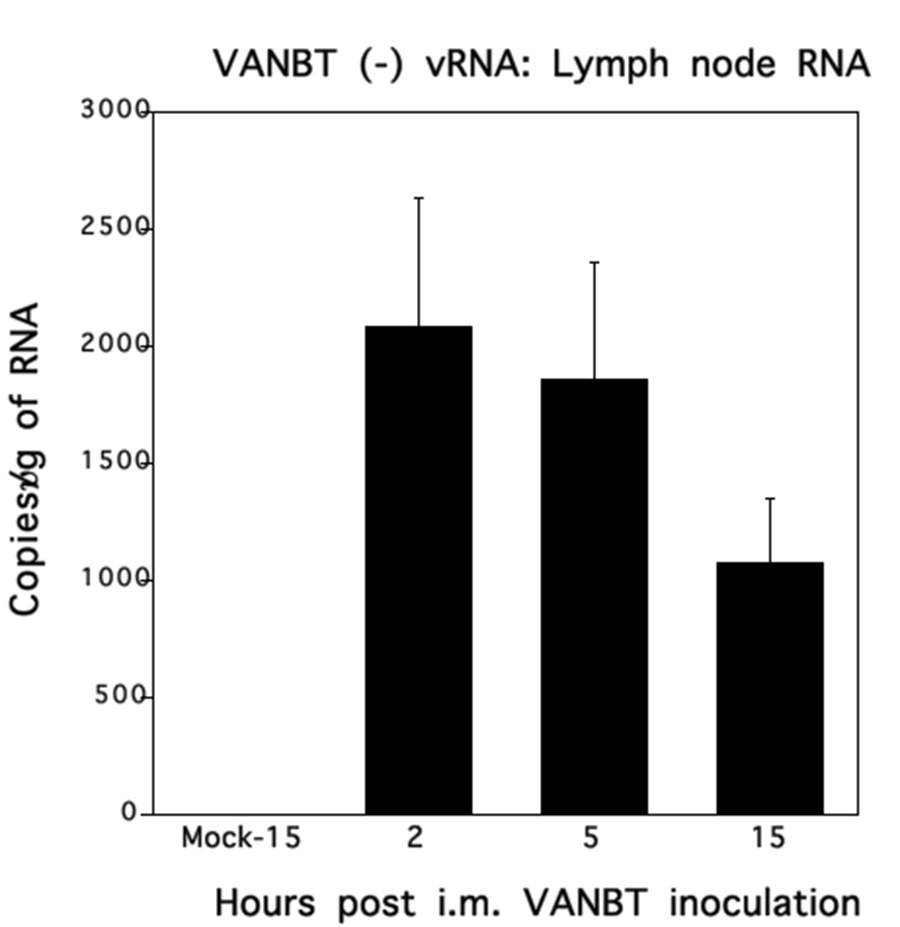

Supplement: S4 Fig — Groups of 3 cotton rats were inoculated i.m. with live 106 TCID50 of VANBT and sacrificed at 2, 5, and 15 h post-inoculation (n = 3/per time point). Two animals inoculated with 1x PBS and sacrificed at 15 h (Mock-15) post-inoculation were shown as control. Inguinal and lumbar lymph nodes, near to the site of injection, were collected from each animal at the indicated time point and (-) vRNA was quantified by qRT-PCR. (TIF) [file pone.0166336.s004.tif]

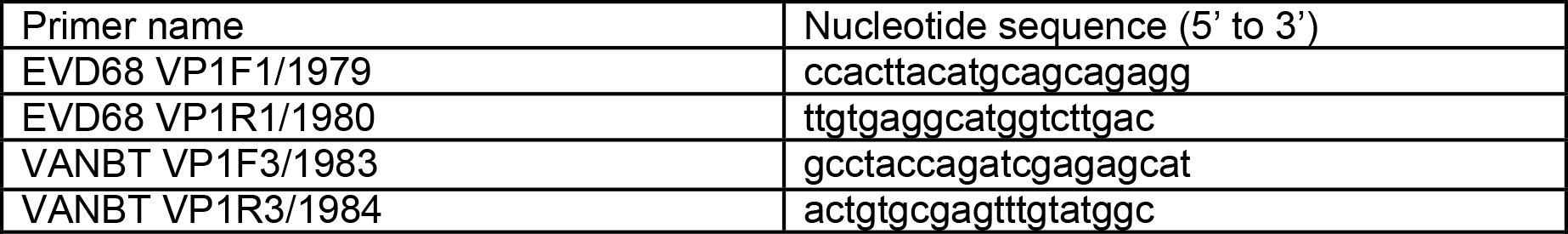

Supplement: S1 Table — (TIF) [file pone.0166336.s005.tif]
